# Neonatal hematological parameters and the risk of moderate-severe bronchopulmonary dysplasia in extremely premature infants

**DOI:** 10.1186/s12887-019-1515-6

**Published:** 2019-04-30

**Authors:** Xueyu Chen, Huitao Li, Xiaomei Qiu, Chuanzhong Yang, Frans J. Walther

**Affiliations:** 1 Department of Neonatology, Affiliated Shenzhen Maternity & Child Healthcare Hospital, Southern Medical University, Shenzhen, China; 2 Department of Pediatrics, David Geffen School of Medicine, University of California Los Angeles, Los Angeles, CA USA; 3 Los Angeles Biomedical Research Institute at Harbor-UCLA Medical Center, Torrance, CA USA

**Keywords:** Extremely prematurity, Bronchopulmonary dysplasia, Hematology, Platelets

## Abstract

**Objective:**

To evaluate the association between hematological parameters at birth and the risk of moderate-severe bronchopulmonary dysplasia (BPD) in a cohort of extremely preterm infants.

**Methods:**

This is a retrospective study of all extremely premature infants admitted to the neonatal intensive care unit, Shenzhen Maternity and Child Healthcare Hospital from January 2016 to May 2018. Extremely prematurity was defined as a delivery at a gestational age ≤ 28 weeks or a birth weight ≤ 1000 g. BPD was diagnosed if oxygen exposure exceeded 28 days and the severity was decided at 36 weeks PMA or discharge. Multivariable analysis was performed to assess the independence of the association between hematological parameters at birth and risk of moderate or severe BPD.

**Results:**

A total of 115 extremely premature infants were analyzed in this study. The median platelet count, neutrophil and monocyte count at birth were significantly higher in infants with moderate-severe BPD compared to infants without BPD (228 vs 194*10^9^/l, *P* = 0.004; 5.0 vs 2.95*10^9^/l, *P* = 0.023; 0.88 vs 0.63*10^9^/l, *P* = 0.026, respectively) whereas the mean platelet volume was significantly lower in infants with moderate-severe BPD than those without BPD (9.1 vs 9.4 fl, *P* = 0.002). After adjusting for covariates, the risk of moderate-severe BPD was independently associated with platelet count≥207*10^9^/l (odds ratio 3.794, 95% confidence interval: 1.742–8.266, *P* = 0.001).

**Conclusion:**

Our findings suggest that hematologic parameters at birth are different in extremely preterm infants who will develop moderate-severe BPD. A higher platelet count at birth may increase the risk of moderate-severe BPD after extremely premature birth.

## Introduction

Bronchopulmonary dysplasia (BPD) affects around 50% of extremely preterm infants [1, 2]. Over the past decades, the survival rate of extremely preterm infants has remarkably increased due to the improvement in perinatal care, such as surfactant therapy and ventilation strategies [3]. Concomitantly, the number of new BPD cases is steadily increasing [4].

The pathogenesis of BPD is largely attributed to the arrested lung development in these extreme preemies [5]. Gestational age at birth is thus of paramount important for the risk of BPD provided that preterm birth interrupts the programmed pulmonary development during intrauterine life [6]. BPD is nearly-always present in survivals from gestations less than 23 weeks (saccular stage of lung development) [7], whereas the risk of BPD in infants born after 30 weeks of gestation steeply declines to 1% [8].

Currently, BPD is thought to begin during the first days of life [9, 10]. The identification of high risk infants therefore facilitates timely intervention to reduce the occurrence of BPD. In preterm infants, hematologic testing is routinely performed at birth to evaluate the neonatal condition. Different types of blood cells play an important role in pulmonary inflammation and associated lung injury in preterm infants [11]. Carlo Dani *et. al* and F. Cekmez *et. al* both reported a high level of mean platelet volume (MPV) in the first days of life is associated with increased risk of BPD in preterm infants [9, 12]. However, the association between hematologic parameters at birth and the risk of BPD in extremely premature infants remains elusive. Therefore, the purpose of this study was to investigate clinical hematologic parameters at birth and their association with moderate and severe BPD in a cohort of extremely preterm infants.

## Methods and materials

### Study design and population

This is a retrospective study performed at the Neonatal Intensive Care Unit (NICU), Shenzhen Maternity and Child Healthcare Hospital from January 2016 to May 2018. This study was approved by the institutional ethic committee. All extremely preterm infants cared for in our center were included in the present study. We excluded neonates due to major congenital anomalies and death prior to the diagnosis of BPD.

### Definition of clinical variables

Extreme prematurity was defined as a delivery at a gestational age ≤ 28 weeks or a birth weight ≤ 1000 g. The diagnosis and severity of BPD in preterm birth was assessed using the consensus definition of National Institute of Child Health and Human Development (NICHD). Briefly, BPD was diagnosed when supplemental oxygen was needed for more than 28 days and the severity was assessed according to the oxygen concentration required at 36 weeks PMA or discharge [13, 14]. (Suspected) Early-onset neonatal sepsis occurring within the first 72 h of life was defined as the following criteria: a positive culture of blood and/or the presence of clinical signs of infection with abnormal chest radiograph profiles, hematological features and maternal risk factors [15].

### Data collection

The following data were retrieved from the electronic medical record, including maternal age, mode of conception, maternal complications such as gestational hypertension and gestational diabetes mellitus (GDM), premature prelabor rupture of membranes (PPROM), chorioamnionitis, small for gestational age (SGA), antenatal steroid, delivery methods, need for resuscitation, gestational age (GA), birth weight (BW), Apgar score, sex, whole blood test at birth, neonatal respiratory distress syndrome (NRDS), surfactant treatment, ventilation mode, patent ductus arteriosus (PDA) and (suspected) early onset neonatal sepsis, intraventricular hemorrhage (IVH), necrotizing enterocolitis (NEC) and pulmonary hemorrhage. Antenatal steroid treatment was considered if at least one dose of dexamethasone was administrated 12 h prior to delivery. Surfactant treatment was recorded if at least one course of surfactant was administrated. Blood testing was performed on Mindray 5390 (Shenzhen, China) using the samples collected within 3 h after birth from the umbilical venous or umbilical artery catheter of the infants.

### Statistics

The sample size calculation was based on the platelet count from our clinical laboratory. At 90% power and α = 0.05, 51 infants in each group would be sufficient to detect a significant difference. Hematologic parameters were expressed as median [interquartile range (IQR)]. The Shapiro-Wilk test was used to evaluate the normality of continuous variables. Unpaired t test or Mann-Whitney U test was adopted to analyze continuous variables, as appropriate. Chi-square or Fisher’s exact test were used to compare categorical data, as appropriate. Multivariate logistic regression was performed to determine the independent risk factors of moderate or severe BPD. The odds ratios (OR) and 95% confidence interval (CI) were calculated in logistic regression analysis. Afterwards, receiver-operator curve (ROC) was applied to calculate the cut-off values to dichotomize the corresponding continuous variables significantly related to the occurrence of moderate or severe BPD in multivariate logistic regression analysis. Finally, univariable logistic regression model was built to find the independent risk factors for the occurrence of moderate or severe BPD and its related morbidities.

### Ethical statement

The Shenzhen Maternity and Child Healthcare Hospital Institutional Ethical Committee (IEC) approved the collection and usage of the clinical information for research purposes and waived the requirement for informed consent (IEC No. [2018]-082).

## Results

**Fig. 1:**
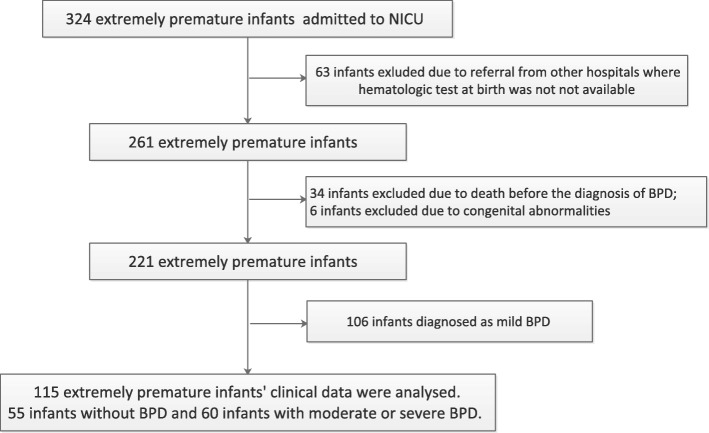
Flowchart of cases selection and analysis. 115 extremely premature infants were enrolled in this study. BPD, bronchopulmonary dysplasia. NICU, neonatal intensive care unit

A total of 318 extremely premature infants were admitted to our NICU during the study period. Diagnosis of BPD was made in 166 (75%) infants in which 106 (48%) infants were categorized as mild BPD and 60 (27%) as moderate-severe BPD (Fig. 1). After applying exclusion criteria, 115 extremely premature infants were included in this study, in which 97 (84%) were born before 28 weeks and 18 (16%) born after 28 weeks with birthweight lower than 1000 g. The median of GA at birth was 26.4 (IQR: 25.1–27.6) weeks. The clinical characteristics are summarized in Table 1.

**Table 1:** Clinical characteristics by bronchopulmonary dysplasia status

Variable	Infants without BPD (*n* = 55)	Infants with moderate or severe BPD (*n* = 60)	*P* value
maternal age, yr	32 (29–36)	32 (29–34)	0.406
conception by ART	4 (7%)	16 (27%)	0.006
GDM	3 (6%)	4 (7%)	0.786
gestational hypertension	9 (16%)	3 (5%)	0.046
PPROM	13 (24%)	24 (40%)	0.061
chorioamnionitis	6 (11%)	2 (3%)	0.015
Intubation at resuscitation	25 (45%)	53 (88%)	< 0.001
antenatal steroid treatment	37 (67%)	49 (82%)	0.076
cesarean section delivery	25 (46%)	10 (17%)	0.001
gestational age at birth, wk	27.3 (26.1–28.6)	25.8 (24.5–26.8)	< 0.001
birth weight, gr	890 (740–980)	770 (687–910)	0.039
SGA	18 (33%)	5 (8%)	0.001
male	26 (47%)	39 (65%)	0.055
NRDS	43 (78%)	54 (90%)	0.081
Mechanical ventilation	22 (40%)	51 (85%)	< 0.001
(Suspected) Early-onset neonatal sepsis	9 (16%)	26 (43%)	0.002
Apgar score at 1 min	7 (5–9)	5 (5–8)	0.041
Apgar score at 5 min	10 (9–10)	10 (8–10)	0.133
surfactant treatment	32 (59%)	53 (88%)	< 0.001
PDA	10 (18%)	35 (58%)	< 0.001
IVH grade 3 or 4	5 (9%)	9 (15%)	0.333
NEC	2 (4%)	3 (5%)	0.541
pneumothorax	1 (2%)	1 (2%)	0.950
pulmonary hemorrhage	2 (4%)	4 (7%)	0.681

Data were displayed as median (interquartile range) or number (percentage). *ART* assisted reproductive technology, *GDM* gestational diabetes mellitus, *PPROM* preterm premature rupture of the membranes, *SGA* small for gestational age, *NRDS* neonatal respiratory distress syndrome, *PDA* patent ductus arteriosus, *IVH* intraventricular hemorrhage, *NEC* necrotizing enterocolitis

Univariable analysis showed that the moderate-severe BPD group had higher rate of conception by ART (27% vs 7%), intubation at resuscitation (88% vs 45%), mechanical ventilation (85% vs 40%), (suspected) early onset neonatal sepsis (43% vs 16%), PDA (58% vs 18%) and surfactant treatment (88% vs 59%, Table 1). In addition, infants with moderate-severe BPD had lower gestational age (25.8 vs 27.3 weeks), birth weight (770 vs 890 g) and 1-min Apgar score (5 vs 7), as well as lower rate of gestational hypertension (5% vs 16%), chorioamnionitis (3% vs 11%), cesarean section delivery (17% vs 46%) and SGA (8% vs 33%, Table 1).

The comparison of hematologic parameters at birth between infants without BPD and with moderate or severe BPD was displayed in Table 2. The platelet count, neutrophils count and percentage, monocyte count and percentage were significantly higher in infants with moderate or severe BPD compared with no BPD infants (228 vs 194 *10^9^/l, *p* = 0.004; 5.0 vs 2.95 *10^9^/l, *p* = 0.023; 49.1% vs 37.4%, *p* = 0.032; 0.88 vs 0.63 *10^9^/l, *p* = 0.026 and 8.0% vs 6.8%, *p* = 0.04, respectively). The mean platelet volume (MPV), basophil percentage and lymphocyte percentage were significantly lower infants with moderate or severe BPD compared with no BPD infants (9.1 vs 9.4 fl, *p* = 0.002, 0.2% vs 0.3%, *p* = 0.011 and 38.5% vs 53.45%, *p* = 0.022, respectively).

**Table 2:** Hematologic features at birth by bronchopulmonary dysplasia status

Variables	Infants without BPD(*n* = 55)	Infants with moderate or severe BPD (*n* = 60)	*P* value
WBC count,10^9^/l	9.12 (6.28–15.89)	11.2 (6.9–20.9)	0.141
RBC count,10^12^/l	4.22 (3.74–4.59)	4.2 (3.9–4.5)	0.523
platelet count,10^9^/l	194.00 (131.00–245.00)	228 (189–259)**	0.004
Neutrophils count,10^9^/l	2.95 (1.68–6.46)	5.0 (2.4–13.1)*	0.023
lymphocyte count,10^9^/l	4.14 (3.17–6.94)	4.1 (3.0–6.7)	0.675
Eosinophil count,10^9^/l	0.12 (0.05–0.18)	0.11 (0.06–0.22)	0.492
Basophil count,10^9^/l	0.02 (0.01–0.06)	0.02 (0.01–0.03)	0.060
monocyte count,10^9^/l	0.63 (0.37–1.12)	0.88 (0.57–1.55)*	0.026
Neutrophil percentage, %	37.40 (26.50–57.30)	49.1 (36.6–63.0)*	0.032
Lymphocyte percentage, %	53.45 (33.35–64.83)	38.5 (29.4–54.3)*	0.022
Eosinophil percentage, %	1.20 (0.60–2.03)	1.1 (0.6–1.6)	0.845
Basophil percentage, %	0.30 (0.10–0.50)	0.2 (0.1–0.3)*	0.011
Monocyte percentage, %	6.80 (4.55–8.63)	8.0 (4.9–10.3)*	0.040
Hb level, g/l	161.00 (138.75–179.75)	156 (143–167)	0.523
Hematocrit, %	50.70 (45.40–56.08)	50 (45–52)	0.171
MCV, fl	120.40 (113.35–128.70)	120 (114–124)	0.549
MCH, pg	38.40 (36.05–39.95)	38 (36–40)	0.582
MCHC, g/l	315.00 (306.75–325.50)	319 (307–326)	0.910
RDW, %	16.5 (15.4–17.8)	15.7 (15.3–16.4)	0.079
MPV, fl	9.4 (9.00–9.85)	9.1 (8.7–9.4)**	0.002
PDW, %	16.75 (16.60–17.10)	16.7 (16.4–17.0)	0.273

Data were displayed as median (interquartile range). *p < 0.05 and **p < 0.01 are compared with no BPD group. *WBC* white blood cell, *RBC* red blood cell, *Hb* hemoglobin, *MCV* mean corpuscular volume, *MCH* mean corpuscular hemoglobin, *MCHC* mean corpuscular hemoglobin concentration, *RDW* red cell distribution width, *MPV* mean platelet volume, *PDW* platelet distribution width

These potential risk factors were subsequently entered into the multivariable regression model. We found that the risk of moderate-severe BPD was independently associated with intubation at resuscitation (OR 4.020, 95% CI: 1.124–14.376, *P* = 0.032), PDA (OR 7.209, 95% CI: 1.980–26.251, *P* = 0.003), (suspected) early-onset neonatal sepsis (OR 6.697, 95% CI: 1.659–27.034, *P* = 0.008) and platelet count (OR 1.011, 95% CI: 1.002–1.021, *P* = 0.022, Table 3).

**Table 3:** Multivariate logistic regression analysis of selected variables associated with BPD

Variables	No BPD (*n* = 55)	Moderate or severe BPD (*n* = 60)	*p*	OR (95% CI)
Gestational age, weeks	27.3 (26.1–28.6)	25.8 (24.5–26.8)	0.100	0.733 (0.506, 1.062)
Platelet count, 10^9^/L	194.00 (131.00–245.00)	228 (189–259)	0.022	1.011 (1.002, 1.021)
(Suspected) Early-onset neonatal sepsis, no	46 (84%)	34 (57%)	–	–
(Suspected) Early-onset neonatal sepsis, yes	9 (16%)	26 (43%)	0.008	6.697 (1.659, 27.034)
PDA, no	45 (82%)	25 (42%)	–	–
PDA, yes	10 (18%)	35 (58%)	0.003	7.209 (1.980, 26.251)
Intubation at resuscitation, no	30 (55%)	7 (12%)	–	–
Intubation at resuscitation, yes	25 (45%)	53 (88%)	0.032	4.020 (1.124, 14.376)

*PDA* patent ductus arteriosus

**Fig. 2:**
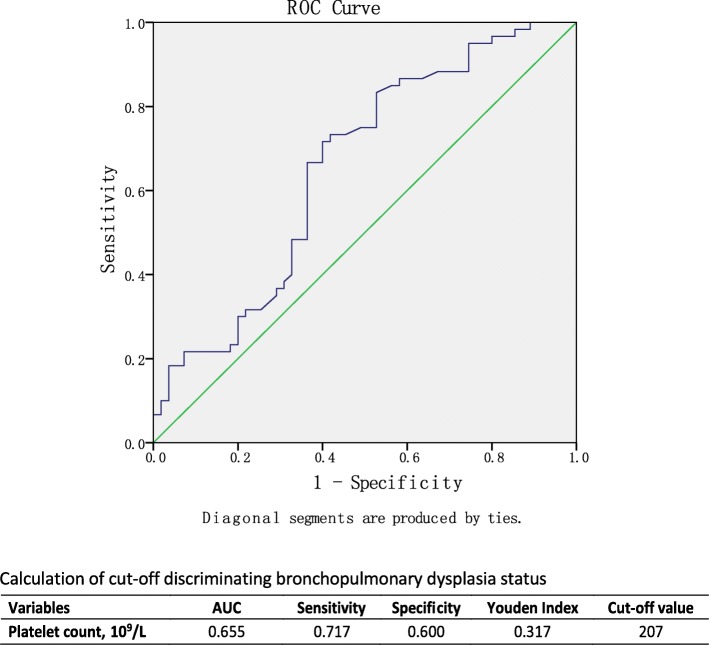
ROC curve of Platelet count with different BPD state and calculation of the cut-off. The cut-off value was calculated to get a maximum Youden’s Index (sensitivity+specificity-1)

Receiver-operator curve was applied to calculate the cut-off value of the significant continuous variables optimally assessing the risk moderate-severe BPD (Fig. 2). A platelet counts of less than 207 *10^9^/l was concluded as the best cut-off value with area under the curve (0.655), sensitivity (0.717), specificity (0.600) and Youden index (0.317). The clinical outcome of this cohort was stratified by the platelet count (Table 4). Besides the effect on the occurrence of moderate and severe BPD, the NICU stay of infants with platelet count > 207 *10^9^/l at birth was slightly longer compared with infants with platelet count ≤207 *10^9^/l at birth (89 (IQR: 62–120) vs 71 (IQR: 50–99), *P* = 0.048).

**Table 4:** Stratification of the Clinical outcome of entire cohort by platelet count at birth

Variables	Platelet ≤ 207*10^9^/l (*n* = 50)	Platelet>207*10^9^/l (*n* = 65)	Odd Ratio	95%CI	P value
Moderate or severe BPD	17 (34%)	43 (50%)	3.794	(1.742, 8.266)	0.001
ROP requiring intervention	12 (24%)	19 (29%)	1.367	(0.589, 3.177)	0.466
Pulmonary hypertension	4 (8%)	7 (10.8%)	1.270	(0.893, 1.179)	0.718
IVH grade 3 or 4	5 (10%)	9 (13.8%)	1.446	(0.453, 4.621)	0.532
NEC	1 (2%)	4 (6.2%)	3.213	(0.348, 29.683)	0.386
Hospital Stay	71 (50–99)	89 (62–120)	–	(−40.020, 10.200)	0.048
Death	0 (0%)	1 (1.5%)	–	–	–

Data were displayed as median (interquartile range) or number (percentage). *ROP* retinopathy of prematurity, *IVH* intraventricular hemorrhage, *NEC* necrotizing enterocolitis

## Discussion

The present study systematically analyzed the hematologic parameters at birth in a cohort of extremely premature infants and further evaluated the association between these features and the risk of moderate or severe BPD. We found that the platelet counts at birth were significantly higher in infants developing to moderate-severe BPD in later life. In addition to the well-known risk factors like intubation at resuscitation, PDA and (suspected) early-onset neonatal sepsis, this study showed that platelet count at birth was also an independent risk factor for the occurrence of moderate-severe BPD. Gestational age, may be owing to the population characters, was identified as a non-independent risk factor.

BPD is a severe complication that leads to increased short- and/or long-term morbidity and mortality. Several hematologic parameters during the first days of life are related to the increased risk of BPD. Palta, M *et. al* found low neutrophil count (< 1*10^9^/l) predicted the BPD severity level (OR: 1.7, 95% CI:1.1–2.7) in very low birth weight (VLBW) infants [16], which is opposite to findings in the current study. Noticeably, a neutrophil count of less than 1*10^9^/l was only detected in 5 infants without BPD and 6 infants with moderate-severe BPD. This discrepancy may thus be attributed to the small sample size in current study. Large studies are needed to validate the predictability of neutrophil count at birth for the risk of BPD.

The association of MPV with the risk of BPD was reported in several studies [9, 12]. Dani *et. al* found that MPV > 11 fl at 24–48 h after birth in infants born earlier than 30 weeks was associated with the occurrence of moderate and severe BPD whereas the MPV and platelet count at birth were comparable in infants with and without moderate-severe BPD [9]. Cekmez *et.al* also found an increased MPV in the first days of life was associated with the development of BPD group in infants born < 34 weeks or with birth weight < 1500 g [12]. However, a slightly lower MPV at birth was found in infants developing into moderate or severe BPD in current study. These discrepancies may be owing to the different study populations.

It is interesting that the platelet counts at birth was associated with the occurrence of moderate-severe BPD. However, the underlying mechanisms remains to be elucidated. Pulmonary inflammation plays a pivotal role in the arrested lung development following extremely preterm birth [4, 5]. In a recent study, Sreeramkumar et al. report that activated platelets initiate inflammation through directing of the neutrophil migration [17]. The elimination of platelets in blood remarkably mitigates pulmonary injury in a mice model of acute lung injury [18]. The lung has been recognized as a site of platelet biogenesis [19], leading to the realization that the immature lung may be a fragile organ in case of inflammation. We thus speculate that the inhibition of platelet activation may ameliorate pulmonary inflammation in extremely premature infants.

A newborn’s platelet count can be influenced by several factors. Infection and inflammation may increase the platelet shortly and then consume a lot. Antibodies generated by maternal immune system under some pathologic condition may also enter the fetal circulation, attack the platelet and lead to decreased platelet count in newborn [20]. In current study, only 4 infants were born with a platelet count less than 100,000/uL, and none of their mothers had platelet count less than 100,000/uL on the day of birth. Besides, maternal complications like preeclampsia and intrauterine growth restriction accompanied by chronic hypoxia may stimulate the generation of reticulocytes and reduce the number and total masses of megakaryocyte, as well as blunt the function of platelet [21]. To exclude these confounding factors, we included early onset neonatal sepsis, chorioamnionitis, SGA and gestational hypertension in our analysis.

The main strength of our study is the great applicability in routine practice. BPD remains a major challenge for perinatologists. The accurate and rapid identification of high-risk infants is of paramount importance for the prevention of BPD. However, our data should be interpreted with care. Besides of the retrospective design, an inclusion bias in our study has incurred because we excluded the infants who died before the diagnosis of BPD was made. These infants may be also at increased risk of moderate or severe BPD due to the intubation in most cases prior to death. Moreover, the cut-off value of hematologic parameters at birth was calculated in a relatively small cohort of extremely preterm infants. Large prospective studies are required to confirm the findings in this study. The function of the platelet was not measured in this manuscript. Besides, it would be interesting to have a look at the continuous platelet count in the first week of life and its predictive value for BPD.

## Conclusion

In conclusion, hematologic parameters at birth are different in extremely preterm infants with moderate-severe BPD. A platelet count > 207*10^9^/l at birth is an independent predictor for the occurrence of moderate-severe BPD.

## Abbreviations

95%CI: 95% confidence interval
ART: Assisted reproductive technology
BPD: Bronchopulmonary dysplasia
BW: Birth weight
ELBW: Extremely low birth weight
GA: Gestational age
GDM: Gestational diabetes mellitus
Hb: Hemoglobin
IVH: Intraventricular hemorrhage
MCH: Mean corpuscular Hemoglobin
MCHC: Mean corpuscular hemoglobin concentration
MCV: Mean corpuscular volume
MPV: Mean platelet volume
NEC: Necrotizing enterocolitis
NICU: Neonatal intensive care unit
NRDS: Neonatal respiratory distress syndrome
OR: Odds ratios
PDA: Patent ductus arteriosus
PDW: Platelet distribution width
PPROM: Premature prelabor rupture of membranes
RDW: Red blood cell distribution width
ROC: Receiver-operator curve
ROP: Retinopathy of prematurity
